# A New Experimental Polytrauma Model in Rats: Molecular Characterization of the Early Inflammatory Response

**DOI:** 10.1155/2012/890816

**Published:** 2012-02-29

**Authors:** Sebastian Weckbach, Mario Perl, Tim Heiland, Sonja Braumüller, Philip F. Stahel, Michael A. Flierl, Anita Ignatius, Florian Gebhard, Markus Huber-Lang

**Affiliations:** ^1^Department of Orthopaedic Trauma, Hand-, Plastic- and Reconstructive Surgery, University Hospital of Ulm, 89075 Ulm, Germany; ^2^Department of Orthopaedic Surgery, Denver Health Medical Center, University of Colorado, Denver, CO 80204, USA; ^3^Institute of Orthopaedic Research and Biomechanics, Center of Musculoskeletal Research Ulm, University of Ulm, 89075 Ulm, Germany

## Abstract

*Background*. The molecular mechanisms of the immune response after polytrauma are highly complex and far from fully understood. In this paper, we characterize a new standardized polytrauma model in rats based on the early molecular inflammatory and apoptotic response. *Methods*. Male Wistar rats (250 g, 6–10/group) were anesthetized and exposed to chest trauma (ChT), closed head injury (CHI), or Tib/Fib fracture including a soft tissue trauma (Fx + STT) or to the following combination of injuries: (1) ChT; (2) ChT + Fx + STT; (3) ChT + CHI; (4) CHI; (5) polytrauma (PT = ChT + CHI + Fx + STT). Sham-operated rats served as negative controls. The inflammatory response was quantified at 2 hours and 4 hours after trauma by analysis of “key” inflammatory mediators, including selected cytokines and complement components, in serum and bronchoalveolar (BAL) fluid samples. *Results*. Polytraumatized (PT) rats showed a significant systemic and intrapulmonary release of cytokines, chemokines, and complement anaphylatoxins, compared to rats with isolated injuries or selected combinations of injuries. *Conclusion*. This new rat model appears to closely mimic the early immunological response of polytrauma observed in humans and may provide a valid basis for evaluation of the complex pathophysiology and future therapeutic immune modulatory approaches in experimental polytrauma.

## 1. Introduction

Trauma is still one of the leading causes of death among people aged 45 and younger. The annual economic burden of direct and indirect costs in Germany alone is estimated to be around 40 billion Euros annually [1]. Due to the heterogeneity of trauma, complex injury patterns, and broad variability of therapeutic options, it is an enormous challenge to collect valid data in prospective or retrospective studies concerning posttraumatic pathophysiological changes and possible treatment options.

Of all polytraumatized patients 86% sustain an injury to the extremities, 69% to the head, and 62% to the chest [2]. In this regard, the effects of a combined injury on the patient are not comparable to that after isolated trauma. Trupka et al. indicated that mortality rises from 7% to 18% when an additional trauma to the chest is present. Musculoskeletal trauma induces the systemic release of diverse “danger molecules” DAMPs (danger-associated molecular patterns) [3] which lead to a pronounced early immunological and inflammatory response [4]. The release of these DAMPs is a tremendous challenge for the immune system in severe trauma as explained by the “danger model” of the immune system (danger sensing, transmission, response, elimination) [3, 5]. As a consequence of the injury severity and the immune status of the patients, the posttraumatic inflammatory response often results in an overindulging and uncontrolled activation of the complement system with an increased release of proinflammatory mediators [6] often resulting in multiple organ dysfunction and death. In this regard, IL-6 has been shown to be elevated at the scene of injury and a positive correlation between these IL-6 levels, the severity of the injury and the increased rate of complications including enhanced mortality has been demonstrated [7, 8]. In addition, increased systemic levels of the anaphylatoxins C3a and C5a in severe polytraumatized patients seem to be correlated with a higher risk of sepsis and a poor clinical outcome [6, 9]. However, up to date, little is still known about the immune response after severe polytrauma on a cellular and molecular basis. In particular, it is still unclear whether the early immune response is qualitatively or quantitatively different after severe polytrauma compared to an isolated injury. Furthermore, there is a lack of pathophysiology-based therapeutic remedies for polytrauma patients to prevent posttraumatic immunosuppression. Therefore, highly standardized experimental polytrauma models are required to define the early complex pathophysiology of severe combined trauma and to examine novel surgical or immunomodulatory treatment options.

In this study, we describe a new polytrauma model in rats and characterize the early systemic and local inflammatory response accompanied by a rapid activation of the complement system similar to that seen in humans.

## 2. Materials and Methods

### 2.1. Animals and Anesthesia

The study protocol was approved by the University Animal Care Committee and the federal authorities for animal research, Tuebingen, Germany. The experiments were performed in adherence to the National Institutes of Health Guidelines for the use of laboratory animals including a total of 352 male Wistar rats (250 g, 10–12 weeks, Jackson Laboratories, Bar Harbour). Anaesthesia was applied i.p. using 75 mg/kg Ketamin (Ketavet, Pfizer Pharma, Karlsruhe, Germany) and 0.4 mg/kg KG Medetomidine i.p. (Dormitor, Pfizer Pharma, Karlsruhe, Germany). Animals which underwent blunt chest trauma were anesthetized with a mixture of 4% sevoflurane (Sevorane Abbott, Wiesbaden, Germany) and 96% oxygen under a continuous flow of 2 L/min and received the aforementioned i.p anesthesia after the chest trauma.

### 2.2. Individual Trauma Models

The rats were randomly assigned to the different trauma groups (each *n* = 6–10). Narcotized rats underwent either a Sham operation (Sham), a blunt bilateral chest trauma (ChT), a blunt bilateral chest trauma and a lower leg (tibia/fibula) fracture with a contra-lateral soft tissue trauma (ChT + Fx + STT), a closed head injury (CHI), a bilateral blunt chest trauma and a head injury (ChT + CHI), or a combination of chest trauma, head trauma, fracture, and soft tissue trauma referred to as polytrauma (PT). Sham animals were anesthetized rats with an incision and surgical closure of the aponeurotic galea. All animals were placed on a heating pad after undergoing the surgical procedures. Continuous reflex status and vital signs of all animals were checked.

Animals subjected to the blunt bilateral chest trauma were narcotized and fixed in a supine position. The trauma was induced by a single blast wave as previously described [10–12]. The traumatic brain injury was induced by a weight drop device inducing a focused blunt injury over an intact skull after 1.5 cm incision of the aponeurotic galea as described in detail elsewhere [13, 14]. After trauma, the incision was closed by a monofil suture. For the right side tibia/fibula fracture a weight drop device (650 g, 13 cm) induced a reproducible closed transverse fracture of the lower leg, as reported in the past [15]. On the contra-lateral gastrocnemic region a soft tissue injury was applied using a weight drop device (170 g, 180 cm) as previously described [16]. Vital signs were documented during the whole experiment and blood gas analysis was performed. Waking the animals after trauma was strictly avoided and anaesthesia was deepened with repeated i.p. injections as necessary. After 2 hrs or 4 hrs, respectively, all rats were sacrificed and the blood and organs harvested.

### 2.3. Blood-, Lung-, and BAL-Fluid Preparation

Whole blood was spun at 500 ×g at 4°C for 10 min, the serum stored at −80°C until final analysis. After death the trachea was dissected and cannulated, the left lung clamped, and the right lung flushed 3 times with 5 mL PBS including 1 : 1000 broad spectrum protease inhibitor (Sigma-Aldrich, St. Louis, MO, USA). The BAL fluids were then centrifuged 450 ×g at (4°C) for 10 min and stored at (−80°C) until analysis. Left lung tissue was filled with formalin after BAL was taken and immediately stored in 10% formalin until evaluation.

### 2.4. Cytokine ELISA

Interleukin (IL)-6, tumor necrosis factor (TNF)-*α*, complement anaphylatoxin 3a (C3a), monocyte chemoattractant protein (MCP)-1 (all BD OptEIA ELISA SET, BD Pharmingen, San Diego, CA), and cytokine-induced neutrophil chemoattractant (CINC) (R&D, Minneapolis, MN, USA) concentrations of BAL-fluids were determined by sandwich-enzyme-linked immunosorbent assay technique (ELISA) according to the manufacturer's recommendation. In serum, IL-6, TNF-*α*, and CINC were detected.

### 2.5. Serum Complement Hemolytic Activity (CH 50)

The activity of the complement system was assessed by CH50 measurements. A dilution series of the samples was made (1/20–1/480) using Tris-buffered saline (TBS) and the diluted samples were incubated for 60 min at 37°C with sheep erythrocytes (Colorado Serum Company, Denver, CO, USA). The hemolytic complement system reaction was stopped using ice-cold TBS and centrifugation at 700 ×g for 5 min. The absorption of the supernatant was measured at 541 nm by spectrophotometry.

### 2.6. Flow Cytometry Analysis

EDTA whole blood (100 *μ*L) was incubated with indicated FITC- or PE-labelled fluorochrome-conjugated monoclonal antibodies at room temperature for 20 min in the dark. Immediately after the incubation period, 2 mL of FACS lysing solution (BD Biosciences) was added to each tube, followed by incubation for 10 min at room temperature in the dark. The tubes were then centrifuged for 5 min at 340 ×g. After centrifugation, the supernatant was removed, and 2 mL of Dulbecco phosphate-buffered saline (DPBS) per tube was added, followed by an additional centrifugation step. After a second washing step, the cell pellet was resuspended in 100 *μ*L of CellFIX Solution (BD Biosciences) for final flow cytometric analysis. Leukocyte populations (neutrophils, monocytes, and lymphocytes) were discriminated by forward/sideward scatter and additional CD45 staining. For each measurement, a minimum of 10,000 events was analyzed. For quantification of C3aR and CRegs (CD35, CD55, and CD59) expression, mean fluorescence intensity (MFI) emitted by the FITC- or PE-conjugated antibodies was calculated by subtracting the corresponding isotype control.

### 2.7. Histological Evaluation

Formalin-fixed lung tissue was dehydrated using ethanol, dissolved with Xylol and embedded in paraffin. Sections of 1 *μ*m were prepared followed by H&E staining. Following that a semiquantitative analysis of the sections including a cell count was performed.

### 2.8. Preparation of PMNs

Whole blood of rats was drawn from the portal vein. Blood was transferred into syringes containing EDTA. Polymorphonuclear neutrophils (PMNs) were isolated using Ficoll-Paque (Pharmacia Biotech, Stockholm, Sweden) gradient centrifugation (340 ×g, 30 min, room temperature). The use of this technique may be responsible for some artificial activation of cells in vivo [17]. For neutrophil isolation, red blood cells (RBCs) were sedimented with dextran and residual RBCs were removed by hypotonic lysis. Neutrophils were resuspended in DPBS and finally diluted at 2 Mio cells per mL. 1 mL aliquots of PMNs were centrifuged at 800 g for 5 min, supernatants were removed, and cells were resuspended in 100 *μ*L of Pharmigen Cell Lysis Buffer (BD Biosciences). After 30 min of incubation on ice, samples were pelleted (16000 ×g, 5 min, 4°C) and supernatants were frozen at −80°C.

### 2.9. Statistics

Results are presented as mean ± SD. A one-way analysis of variance (ANOVA) followed by the Student-Newman-Keuls test as a post hoc test for multiple comparisons was performed to determine significant differences between experimental means. A *P* value of less than 0.05 was considered statistically significant.

## 3. Results

### 3.1. Hematologic Findings

The blood gas analysis (2 and 4 hrs after injury) was indicative of stable Hemoglobin (Hb) and Hematocrit (Hct) values in this polytrauma model. The Hb values did not significantly differ in Sham and PT animals (Sham 2 h 16.5 ± 0.4 g/dL versus PT 2 h 16.2 ± 0.4 g/dL Sham 4 h 16.5 ± 0.32 g/dL versus PT 4 h 15.4 ± 0.4 g/dL; data not shown). The pO_2_ levels stated no hypoxic state in the anaesthetized animals (Sham 4 h 87.7 ± 2.0 mmHg versus PT 4 h 90.0 ± 3.6 mmHg, data not shown).

### 3.2. Systemic Inflammatory Response following Trauma

**Figure 1:**
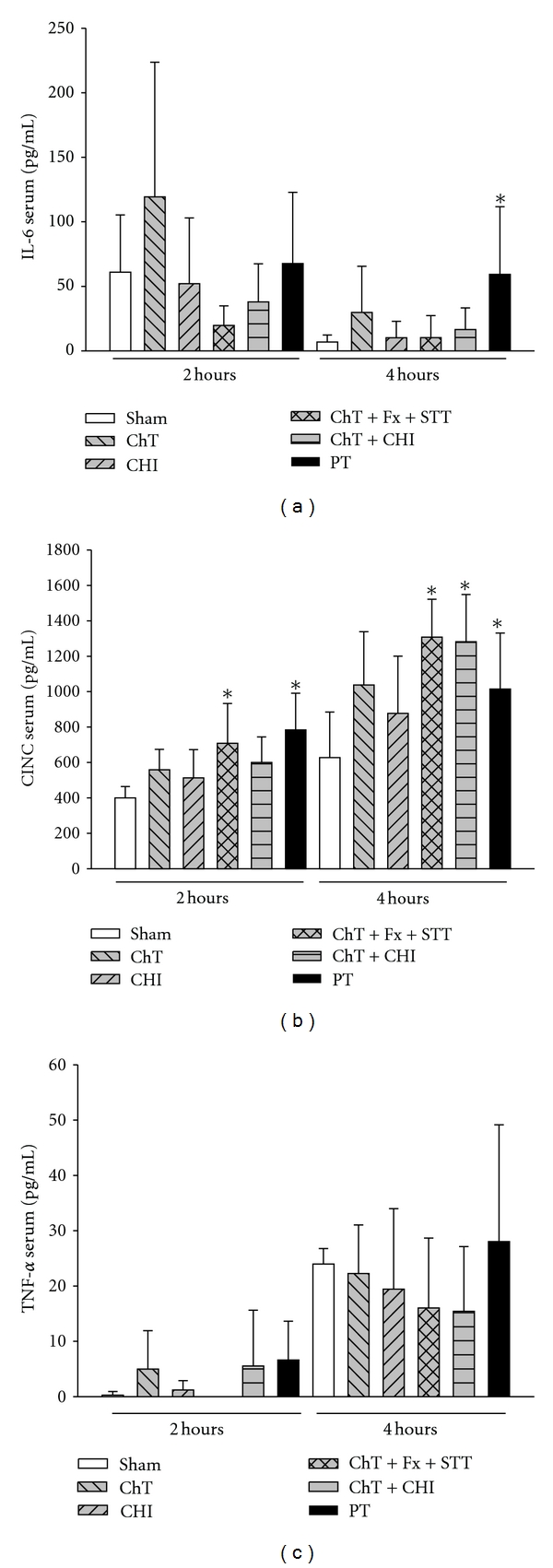
*Systemic inflammatory response following trauma*, (a) shows the systemic IL-6 (pg/mL) serum levels in Sham versus PT animals 2 and 4 hrs after trauma, (b) the CINC (pg/mL) serum concentrations in Sham versus PT animals 2 and 4 hrs after trauma and (c) the TNF-*α* (pg/mL) serum levels in Sham versus PT animals 2 and 4 hrs after trauma. All data are presented as mean ± SD. *P < 0.05. *n* = 6–10 rats/group.

As early as 2 hrs after injury, systemic IL-6 levels were not significantly increased in PT animals compared to the sham group. However, 4 hrs after polytrauma there was a significant increase in the IL-6 concentrations (Figure 1(a)). Similarly, CINC serum concentrations were significantly enhanced in ChT + Fx + STT, ChT + CHI, and PT groups compared to the Sham group 4 hrs after injury (Figure 1(b)). Systemic TNF-*α* levels were rather unchanged in all groups up to 4 hr after injury (Figure 1(c)).

### 3.3. Local Inflammatory Response following Trauma

**Figure 2:**
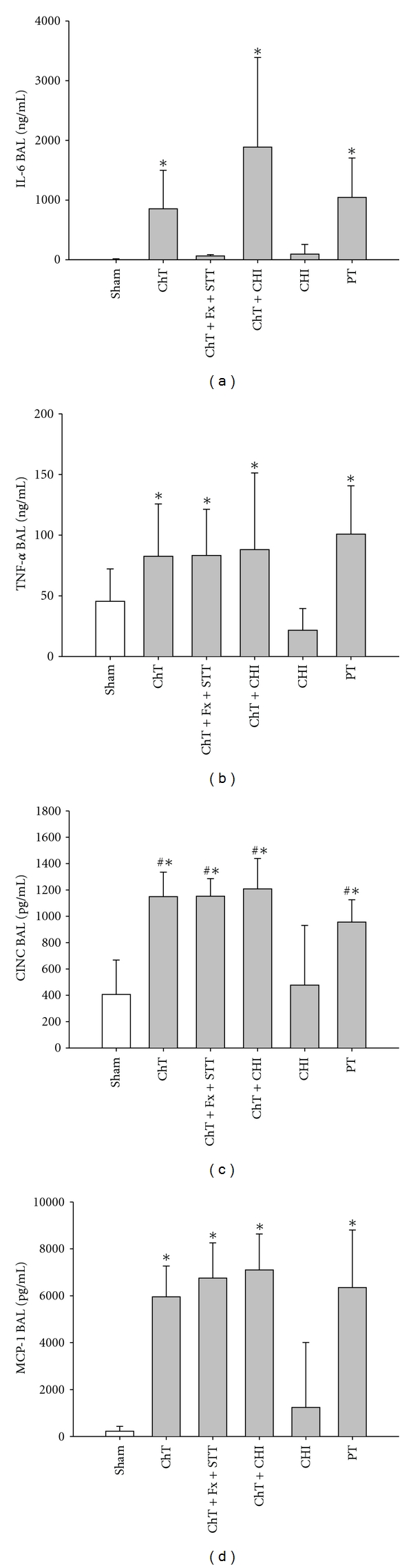
*Local inflammatory response following trauma, *(a) shows IL-6 (ng/mL) BAL-Fluid levels in Sham-, ChT-, ChT + Fx + STT-, ChT + CHI-, CHI- and PT-rats 4 hrs after trauma, (b) TNF-*α* (ng/mL) BAL-Fluid levels in Sham-, ChT-, ChT + Fx + STT-, ChT + CHI-, CHI- and PT-rats 4 hrs post trauma, (c) CINC (pg/mL) BAL-Fluid concentrations in Sham-, ChT-, ChT + Fx + STT-, ChT + CHI-, CHI- and PT-rats 4 hrs after trauma and (d) MCP-1 BAL-Fluid levels in Sham-, ChT-, ChT + Fx + STT-, ChT + CHI-, CHI- and PT-rats 4 hrs after trauma. All data are presented as mean ± SD. *P < 0.05 to Sham; ^#^ *P* < 0.05 significant to CHT. *n* = 6–10 rats/group.

In addition to the systemic inflammatory response, the local inflammatory changes were investigated with focus on the lungs. The local concentrations of IL-6 in the BAL fluids were increased in PT, ChT + CHI and ChT rats 4 hrs after injury compared to Sham-treated rats (Figure 2(a)). Four hours after trauma, the local TNF-*α* levels were increased in all trauma groups except CHI alone (Figure 2(b)). CINC and MCP-1 levels in BAL-fluids were significantly increased in all groups compared to Sham and CHI animals 4 hrs after injury (Figures 2(c) and 2(d)).

### 3.4. Histological Changes in Lung Tissues after Trauma

**Figure 3:**
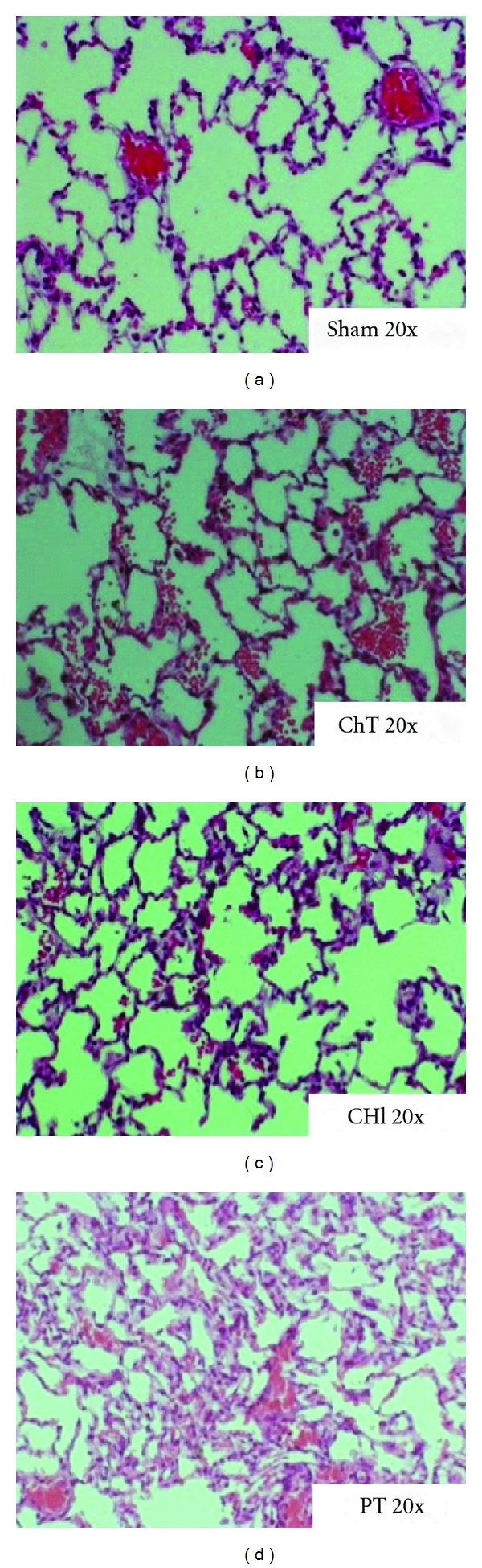
*Histological changes in lung tissues after trauma, *H&E stained lung tissue sections of Sham-, ChT-, CHI and PT-rats analysed by light microscopy with 20x amplification.

Early morphological changes were assessed on H&E stained lung tissue sections analyzed with 20x magnification. Lung sections of the control animals showed physiological lung parenchyma identical to the morphology seen in the traumatic brain injury (CHI) group. After bilateral blunt chest trauma plentiful erythrocytes could be found intra-alveolar, intra-bronchial and sub-pleural. In addition an increased number of alveolar macrophages, some damage to the alveolar wall and tissue edema were found. Similar qualitative morphological changes could be detected in PT-animals but all in a markedly aggravated intensity (Figure 3).

### 3.5. Complement Response after Trauma

**Figure 4:**
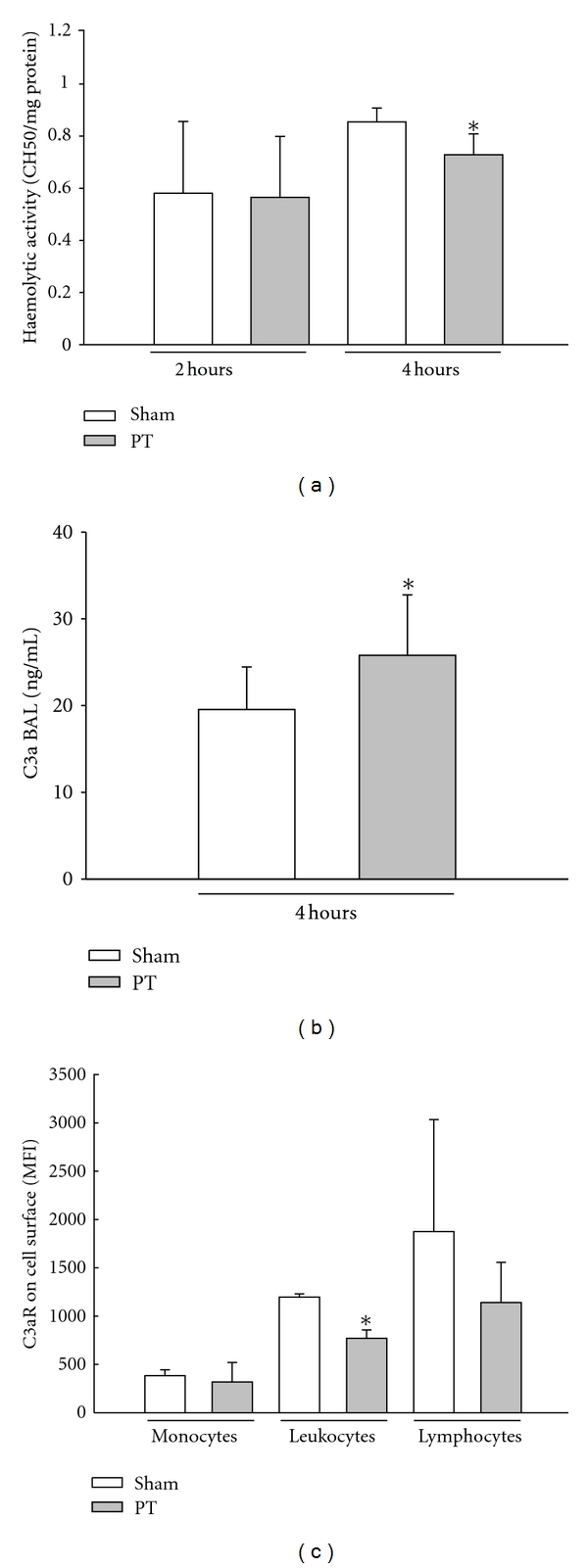
*Systemic complement response after trauma, *(a) presents the hemolytic activity (CH50/mg) on Sham- and PT-rats 2 and 4 hrs after trauma, (b) the C3a (ng/mL) BAL-Fluid levels in Sham- versus PT-rats 2 and 4 hrs post trauma and (c) the C3aR Expression (MFI) on Monocytes, Neutrophils and Lymphocytes in Sham- and PT-rats 4 hrs after trauma. All data are presented as mean ± SD. *P < 0.05. *n* = 6–10 rats/group.

**Figure 5:**
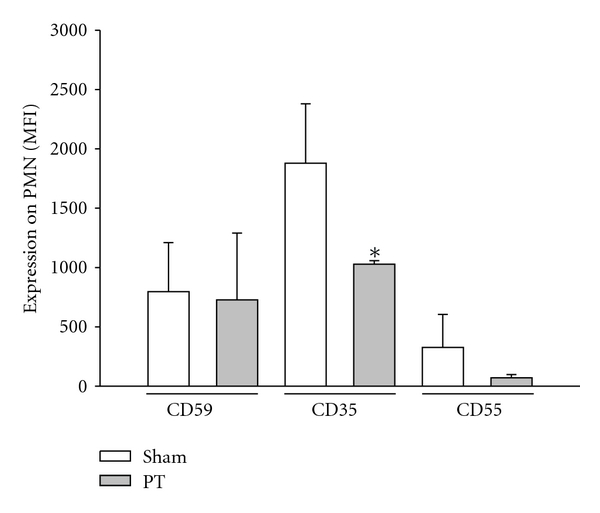
*Complement regulatory response after trauma, *CReg Expression (MFI) CD59, CD35 and CD55 on neutrophils (PMNs) in Sham- and PT rats 4 hrs after trauma. All data are presented as mean ± SD. *P < 0.05. *n* = 6–10 rats/group.

The trauma-induced changes of the complement system were first screened by CH50 testing (Figure 4(a)). After 2 hrs there was a minimum decrease in CH50 values in the PT-group compared to the Sham group, but after 4 hrs this difference reached statistically significance, indicating systemic complement activation. Focusing on the central complement activation product C3a, BAL-fluid levels of C3a were significantly increased in PT animals compared to Sham animals 4 hrs after trauma (Figure 4(b)). The related C3aR revealed a significant loss of surface expression on neutrophils isolated 4 hrs after polytrauma, whereas the C3aR profile remained rather unchanged on monocytes and lymphocytes (Figure 4(c)). Protectin, also known as CD59, exhibited no difference in the expression on neutrophils after trauma (Figure 5). In contrast, complement receptor 1 (CD35) was significantly decreased in PT animals versus Sham rats (Figure 5). A slight but insignificant decrease in expression of CD55 could be detected on neutrophils in PT-rats (Figure 5).

## 4. Discussion

The clinical and molecular danger management after polytrauma is rather complex and often associated with fatal complications. However, the resulting immune and inflammatory response is still in the dark. The aim of this study was to establish a highly standardized and reproducible rodent polytrauma model capable of closely mimicking the posttraumatic inflammatory response found in humans.

Patients at the scene are dying mostly based on severe brain injury or massive blood loss following an injury to a large blood vessel. In contrast, mortality of patients in the hospital several days after major trauma is often associated with a severe systemic inflammatory response (SIRS) and resulting systemic changes such as coagulopathy and complementopathy, breakdown of physiological barriers, impairment of the immune defense, and finally organ dysfunction and failure [6]. The proinflammatory cytokines (e.g., IL-6, IL-8, etc.) and complement anaphylatoxins (e.g., C3a, C5a) were experimentally and clinically proposed to be crucially involved in the early damage response after severe trauma and somehow indicative of the degree of tissue damage and clinical outcome [6, 9]. In the cecal ligation and puncture (CLP) sepsis model in rodents, the complement activation product C5a has been shown to contribute significantly to the development and progression of the systemic inflammatory response. Furthermore, blockade of the C5a-C5aR interaction was associated with improved function on a molecular, cellular, and organ-level and finally resulted in a beneficial outcome [18–20]. However, it is rather unclear if a similar immune modulation would provide some advantageous effects in the setting of multiple injuries. This needs to be investigated further using this new polytrauma model. In the context of the complexity of the polytrauma danger response and the lack of effective therapeutics in the early posttraumatic phase, it is somehow surprising, that despite highly effective anaesthesia protocols there is a worldwide lack of valid rodent models closely simulating multiple injuries of humans. The present blunt trauma model was designed to combine clinically important injury patterns. The single traumata used has been well established and are highly standardized and reproducible in rodents [11–13, 15, 21, 22]. Upon adjustment to the combined injury application, this model provides the unique opportunity to study the impact of an individual trauma and different trauma combinations on the following immune response. A lethality of up to 20% could be detected in this model which reflects the lethality of polytraumatized humans (annual report of the German trauma register 2009). Care has been taken to first design a hemodynamically stable polytrauma model to rule out hemorrhagic shock effects on the inflammatory response. According to Sauaia et al. hemorrhage following traumatic injury accounts for 30–40% of deaths [23]. This could be excluded by BGA measurements showing stable haemoglobin and hematocrit values. However, hemorrhage/shock as well as abdominal trauma certainly represents certainly a major trigger of the inflammatory response [24]. Thus, pressure-controlled hemorrhage or CLP may be included in this model in future studies to discriminate their contribution to the inflammatory response as well.

The early systemic inflammatory response in the present multiple injury model was reflected by enhanced serum levels of IL-6 and CINC which are also enhanced in polytrauma patients [25, 26] and somehow associated with the injury severity and predictive of the clinical outcome. In comparison to Sham-treated animals, the systemic TNF-*α* levels of PT littermates were rather unchanged in the early posttraumatic phase. In contrast, TNF-*α* levels in multiply injured patients were found to be increased early after trauma [27] but rather irrelevant for the prediction of injury severity or outcome. In ex vivo experiments, even a decrease in TNF-*α* levels in stimulated whole-blood samples from polytraumatized patients 2–4 hrs after trauma was reported in relation to healthy donor samples [28].

The local inflammatory polytrauma response with focus on the lungs as “the engine of multi-organ dysfunction” [2] was reflected by increased concentrations in BAL fluids of cytokines and chemokines early after trauma. Similar chemokine profiles have been reported for blunt chest trauma alone by our group [29]. In the clinical setting of blunt chest trauma, there is a lack of data in BAL-fluids [30] but a distinct increase of these cytokines in whole blood with an overall correlation with lung injury severity [8, 26]. Present histological analysis of polytraumatized rats revealed severe intra-alveolar, intrabronchial, and subpleural hemorrhage as well as the presence of interstitial oedema, atelectasis, and an increased amount of alveolar macrophages. These changes have been described in experimental blunt chest trauma alone [22], but in PT animals an aggravation of the histological alterations is evident. The pathophysiological reasons for this enhanced lung injury are rather speculative and might be due to the accompanied brain injury with subsequent disturbances of the neurohormonal-stress-inflammation axis or due to a specific mediator release by the fracture. Fremont et al. described that polytraumatized patients with systemically increased plasma levels of TNF-*α*, IL-8, or IL-10 are more likely to develop ARDS [31].

The complement response globally screened by CH50 changes was unaltered early after polytrauma. However, 4 hrs after multiple injuries, a decent but significant decrease in CH50 was observed. It is noteworthy that already a reduction in CH50 by 10% can be considered as evidence of significant systemic complement activation. These findings are supported by recent reports showing complement activation after major trauma in humans [32].

Complement regulation after severe tissue trauma has been recently addressed by our group, describing a specific leukocyte expression profile of the complement regulatory proteins (CRegs) CD55, CD59, and CD35 early after polytrauma. Whereas CD59 and CD55 were rather unchanged, CD35 expression on neutrophils in the PT versus Sham-group was significantly reduced. The dynamic of CReg expression beyond the 4 hr observational period remains to be seen. However, based on the extremely heterogenous patient groups and the complexity of the pathophysiological reaction, the apoptotic response has been reported rather controversially in polytrauma patients [33, 34]. Guo et al. described during sepsis induced by CLP a C5a-induced decrease of the neutrophil apoptosis rate along with increased levels of Bcl-XL and decreased levels of Bim [35]. Addressing the posttraumatic regulation of the reduced neutrophil apoptosis rate further mechanistic studies need to be performed in the present novel polytrauma model.

The present study has some limitations. During the short observation period, 4 hrs maximum according to the Animal Care Committee protocol, after injury the organisms may be incapable to building up the full inflammatory response especially in the absence of shock symptoms. In addition, the applied traumata and early posttrauma phase occurred in deep anaesthesia, which certainly exclude an additional stress-load seen in reality. On a technical level, although performed within 7 min, the traumata could not be applied simultaneously as it usually occurs in reality.

The role of each single trauma on the pathophysiology of polytrauma leading to SIRS, Sepsis, and MOF is still unknown. However, each isolated injury is survivable, the combination may become lethal. The patient's prognosis and the outcome mainly depends on the presence of the traumatic brain injury and the associated secondary brain damage [36].

In summary, this study is—to our knowledge—the first to demonstrate very early inflammatory changes in a highly standardized, reproducible, hemodynamically stable polytrauma rodent model, mimicking most important injuries of polytraumatized patients excluding hemorrhagic shock. The present polytrauma model may therefore represent the basis for further investigation of the pathophysiology of polytrauma and the evaluation of early therapeutic interventions.
